# On the function of object cells in the claustrum—key components in information processing in the visual system?

**DOI:** 10.3389/fncel.2015.00443

**Published:** 2015-11-05

**Authors:** John Smythies

**Affiliations:** Center for Brain and Cognition, University of California, San DiegoLa Jolla, CA, USA

**Keywords:** claustrum, “object” neurons, synchronization, timing mechanisms, “binding” problem, McGurk effect, Visual Index Theory

A recent paper by Jankowski and O'Mara (2015) throws new light on the function of the claustrum. These authors took recordings from 874 claustral neurons in unanesthetized behaving rats that showed responses probably anchored to visual stimuli. These fell into three categories—“place” neurons (38) that responded to the location of the rat, “boundary” neurons (23) that responded to boundaries closing the rat's environment, and “object” neurons (48) that fired only in response to the existence of a specific object in the environment. All three types of response showed with good within-session stability. Of particular interest in the present context are the object cells. These code the position in space of an object in the environment and not its particular properties (e.g., shape, texture, color). They also follow an object when it moves in the environment. Some object neurons can follow multiple objects (up to 3). Object neurons are also found in the subiculum, and in the anterior entorhinal, perirhinal, and anterior cingulate cortices with which areas the claustrum has extensive connections.

The authors suggest that this system processes dynamic information about spatial features of the environment relating to the location in space of the organism, and the presence of important features in the environment such as boundaries and objects. The claustrum then supplies this information to the wider brain systems in the hippocampus and related cortices so as to facilitate the moment-to-moment control of behavior.

The question this opinion piece addresses is how does this new data relate to a current hypothesis as to the function of the claustrum first put forward by Crick and Koch (2005) and elaborated by Smythies et al. (2012, 2014)? This hypothesis suggests that the claustrum integrates the sensorimotor mechanisms that control behavior via competitive “winner-takes-all” synchronized gamma oscillations in the cortico-claustral circuits by the Pearson system. This system states that, if two cortical areas A and B connect by synchronized oscillations, and, if A connects to claustral area C and B connects to claustral area D, then C, and D will be connected by synchronized oscillations. However, if A and B are not so connected, then C and D will not be connected either.

One “low level” feature of our original hypothesis is that the “binding” of disparate color, shape and movement sensations in the perception of a unitary perceptual object may mediated by this synchronization of gamma oscillations. Another “high level” feature of this hypothesis, also using competitive synchronized oscillations, is that circuits linking the claustrum with higher cortex mediate voluntary decisions involving the selection of particular behaviors in response to complex sensory and multisensory inputs.

Four recent papers are relevant to this hypothesis.

- Firstly, from experiments with brain slices, Orman (2015) has reported that the claustrum has an intrinsic excitatory connectivity, manifesting as spontaneous synchronized burst discharges, that is constrained in approximately rostro-caudal laminae, with minimal cross-communication between laminae. This is compatible with our hypothesis. The mainly rostro-caudal laminae provided by the Pearson mechanism do not interconnect i.e., one active group ABCD does not interact with another active group EFGH.
- Secondly, on the basis of a series of psychophysical experiments, that paired the perception of color with motion or color with orientation, Zeki (2015) has proposed that the brain is a massively asynchronous organ and has no central (master) clock that resets the activity in each of its parallel systems. His experiments showed, for very brief exposures to the stimulus, that we perceive color 40 ms before we perceive form and 80 ms before we perceive motion. Thus, in the visual system color, form and motion are processed independently resulting in an asynchronous behavioral output from each independently. *In other words, under these circumstances, there is no “binding” process involved in visual perception at this level*. However, for longer exposures, and in normal on-going perception, this effect is not seen, and the color, shape and movement of an object are seen as bound together. The reason for this difference is not at present understood (Zeki, 2015). To explain why this asynchronization is seen with, and only with, very short stimulus exposures, we can suggest that three visual pathways (color, shape and motion) above are faster than the claustral pathway (2) listed above that carries the information that there is an object out there. Consequently, for very brief stimulus exposures, there is not enough time for pathway 2 to deliver its message—so “binding” cannot be completed. This suggests that experiments could be done to monitor the bioelectrical activity of object cells during the rat's perception of objects.
- Thirdly, employing advanced multivoxel fMRI pattern analysis techniques, Erez et al. (2015) have produced strong evidence that the construction of the form of visual phenomenal objects in higher visual cortex (especially perirhinal cortex) *is effected by an explicit conjunctive coding mechanism (based on an elaborate Hubel and Wiesel's hierarchical system)*. However, they did not investigate how the color and motion aspects of the visual triad are constructed, nor how these aspects are interrelated with each other and with the form system. So their findings are not directly relevant to the binding problem. These experiments should be repeated and adapted in a study of interactions between form, color and motion.
- Fourthly, Baizer et al. (2014) have found structural discontinuities in the anatomical structure of the claustrum that is minimal in some primates but marked in cetaceans. The authors conclude that this fact argues against the hypothesis put forward by Crick and Koch (2005) and by Smythies et al. (2012) that intraclaustral processing of information is important. Instead, they suggest that each functional subdivision of the claustrum simply contributes to the function of its cortical partner. However, it can be argued that their technique only reveals where the claustral neurons are located, not how they are interconnected. In particular their hypothesis fails to account for the extensive evidence that the claustrum functionally interconnects many systems (Minciacchi et al., 1985; Zhang et al., 2001; Nunn et al., 2002; Emrich et al., 2006; Kavounoudias et al., 2008; Remedios, 2012; Ishizu and Zeki, 2013). In particular Torgerson et al. (2015) recently reported that network theoretical analyses show that the claustrum is a primary contributor to global brain network architecture especially between the frontal lobe and cingulate regions. They conclude that this shows that the claustrum functions as the putative “gate keeper” of neural information for consciousness awareness.

It is possible that the new data by Jankowski and O'Mara might answer Zeki's question. If the information—that the experiences of color, shape and movement of a visual object come from one single object—is not supplied by synchronization of the direct sensory input, might it be supplied instead by activation of particularly located “object” neurons in the claustrum (and other “object” responsive neurons in related cortex)? The “object” neurons might play the role in modulating spatially guided behavior in the manner suggested by Jankowski and O'Mara (2015) and also, in addition, play a role in “binding.” This entails that —

1. During the visual perception of an object, the information as to the particular properties of that object (i.e., its color, shape and movement) is transmitted in parallel and processed independently in the three pathways concerned as Zeki (2015) reports—with little “cross talk” (Orman, 2015). This lack of cross-talk entails that the system gets no information that these stimuli come from a single object and
2. The information that these messages originate in a single object is carried in parallel by the claustral object neuron system operative in that particular location.

My hypothesis involving object neurons in the claustrum has close relations to the Visual Index Theory put forward by Pylyshyn (2001). In that, he suggests that, in perception and cognition, the brain does not operate primarily by noting first the properties of objects and then coordinating these into objects. He suggests instead that, in real life, information access to the visual world (attention) is allocated first mainly, but not entirely, to objects. This involves the primary detection and tracking of objects. He says:

“It may be that we detect *objecthood* first and determine location the way we might determine color or shape—as a property associated with the detected objects. If this is true then it raises some interesting possibilities concerning the nature of the mechanisms of early vision. In particular, it adds further credence to the claim that we must have a way of referring directly to primitive visual objects without using a unique description under which that object falls.”

The discovery of object neurons in the claustrum may well have implications for Pylyshyn's theory.

More evidence relating to the role of synchronized oscillations in neurocomputation has been provided by studies of the McGurk effect. This effect is a species of audio-visual speech sensory integration in which similar, but subtly different, phonemes are presented by the auditory and visual routes. The result is that a third phoneme intermediate between these two is actually heard. In McGurk-negative people this process is faulty and the subject experiences two different phonemes by the auditory and visual routes that do not blend. Fingelkurts et al. (2003) have studied this phenomenon by the brain operational activity EEG/MEG technique. This yields information about the level of synchrony in the different brain operations involved. These workers define multisensory integration in this instance as an emergent process, which combines unimodal signals into a new multimodal representational percept. They showed that the McGurk effect in normal people is accompanied by moment-to-moment metastable synchronization in the beta and alpha ranges of the on-going changes of brain. Moreover, in McGurk-negative subjects this synchronization is actively suppressed. Unfortunately there is no currently information whether alpha and/of beta range EEG activity operates in the claustrum.

One hypothesis re “binding,” that has been widely supported, is that gamma oscillations provide a “clock” for precise temporal encoding and “binding” of signals about stimulus features across brain regions. To test this Burns et al. (2011) computed phase and frequency trajectories of gamma-band bursts, using time-frequency analysis of LFPs recorded in macaque primary visual cortex (V1) during visual stimulation. The authors define the term “clock” to indicate a signal that supplies a regular deterministic structure for time-dependent computation. Their data were compared with simulations of random networks and clock signals in noise. The authors report that gamma-band bursts in LFP data were statistically indistinguishable from those found in filtered broadband noise. They concluded that V1 local field potential data did not contain clock-like signals. However, they suggest that a noisy gamma signals could still perform timing functions in neurocomputation. This system could act as a resonant stochastic filter that could operate as a transient synchronizing pulse that could synchronize different gamma-activated networks to fire simultaneously.

If the “low” level action of the claustrum in binding the visual input is carried out by the action of “object” neurons rather than by integrating gamma oscillations, then the action of the mechanism we have proposed for the integrative action may be limited to the “high” level that refers to the postulated competitive interactions at the highest sensorimotor level of synchronized oscillations in multisensory, cognitive and limbic cortico-claustral circuits for access to the executive motor cortex (Smythies et al., 2012, 2014) in the control of voluntary behavior. For example, during a piano recital, the pianist's brain needs constantly and dynamically to integrate complex auditory, visual, somatosensory, and limbic signals and motor instructions so that she can produce a smooth flow of music. The claustrum may play a key role in this process.

## Conflict of interest statement

The author declares that the research was conducted in the absence of any commercial or financial relationships that could be construed as a potential conflict of interest.
